# Primary Intestinal Lymphangiectasia as a cause of secondary combined immunodeficiency: case study and literature review

**DOI:** 10.1016/j.clicom.2026.05.002

**Published:** 2026-05-15

**Authors:** Rebecca M. Hall, Laurie-Ann Panton, Linda Ineus, Kyung Rae Kim, Ashley T. Nguyen, Kailey E. Brodeur, Pui Y. Lee, Victor L. Fox, Jason Shapiro, Irina Gorbounova, David P. Hoytema van Konijnenburg

**Affiliations:** a Division of Immunology, Boston Children’s Hospital, Harvard Medical School, Boston, MA, USA; b Hasbro Children’s Hospital/ Rhode Island Hospital, and Warren Alpert Medical School of Brown University, Providence, RI, United States; c Division of Vascular and Interventional Radiology, Boston Children’s Hospital, Harvard Medical School, Boston, MA, USA; d Division of Gastroenterology, Hepatology and Nutrition, Boston Children’s Hospital, Harvard Medical School, Boston, MA, USA

**Keywords:** Intestinal lymphangiectasia, Immunodeficiency, Protein-losing enteropathy, Waldmann’s disease, Hypogammaglobulinemia, Cryptosporidium

## Abstract

Primary intestinal lymphangiectasia (PIL) is a rare disorder characterized by the dilation of intestinal lymphatic vessels, resulting in lymph loss. We present the case of an eleven-year-old male, referred to immunology after recurrent *Cryptosporidium* infections. PIL was confirmed after stool studies, endoscopy and imaging. Clinical immunophenotyping revealed combined immunodeficiency, with severe hypogammaglobulinemia (IgG: 212 mg/dL) and T cell lymphopenia (CD4^+^ count: 172 cells/μL; CD8^+^ count: 208 cells/μL). After treatment with subcutaneous immunoglobulin and trimethoprim/sulfamethoxazole prophylaxis, immune parameters remained suboptimal, and dietary strategies were implemented. This intervention resulted in improvement in his IgG level, rising to 1114 mg/dL, although T cell lymphopenia remained. We discuss the clinical course of this patient and provide an overview of the published literature regarding immunodeficiency associated with PIL. We conclude that PIL should be considered as a cause of secondary combined immunodeficiency in the absence of an alternative explanation in both adult and pediatric patients.

## Introduction

Primary intestinal lymphangiectasia (PIL), also referred to as Waldmann’s disease, is known for the dilation of the intestinal lymphatic system. The dilation, sometimes even rupture, of the lymphatic vessels causes the leakage of lymph through the gastrointestinal tract. This leakage can subsequently cause hypoproteinemia, hypogammaglobulinemia, and lymphopenia [1]. The disorder is frequently framed in gastrointestinal terms because of its classic presentation of diarrhea, edema, and malabsorption, but the lymphatic leakage also can cause significant immunologic dysfunction, sometimes mimicking primary immunodeficiencies [2,3]. Intestinal lymphangiectasia (IL) can be induced by risk factors such as heart surgery, chemotherapy, infection, or toxic materials known to trigger lymphatic changes, however, PIL occurs congenitally in the absence of causative factors. Diagnosis requires endoscopic visualization of abnormal mucosa and histologic confirmation through biopsy. The extent of the lymphangiectasia can be seen via capsule endoscopy, MR lymphangiography, or whole-body MRI [4]. Here, we present a case of an eleven-year-old male with PIL who presented to outpatient immunology for a recurrent *Cryptosporidium* infection, then found to have combined immunodeficiency. We provide a review of the literature, demonstrating the prevalence of a secondary combined immunodeficiency phenotype in PIL.

### Case study

An eleven-year-old male had an unremarkable past medical history until the development of a recurrent *Cryptosporidium* infection. The patient was born healthy at term via C-section with growth consistently at the 50th percentile. Early childhood was notable for frequent episodes of croup, resolving by age five, and a few ear infections. Prior to his immunologic workup, the patient experienced four weeks of diarrhea and intermittent emesis, two weeks of abdominal bloating, and endorsed generalized, non-radiating abdominal pain occurring approximately four times a week. The pain worsened with consumption of dairy products and was sometimes associated with decreased appetite. Physical examination was notable for abdominal distension, ascites, and pleural edema. Initial laboratory investigations revealed leukocytosis with WBC 12.1 × 10^9^ (range 4.4–11 × 10^9^/L), thrombocytosis (420 × 10^9^, range 168–382 × 10^9^/L), hypoalbuminemia (1.8 g, range 3.1–4.8 g/dL), and hypoproteinemia (total protein <3.0 g (range 6.1–8.0 g/dL), along with a mildly elevated fecal calprotectin of 73.9 mg/kg (range 27.1–49.9 mg/kg). Stool gastrointestinal PCR identified *Cryptosporidium*. Serum tissue transglutaminase IgA (TTG IgA) was elevated at 55.8 U/mL (range 0.0 – 14.9 U/mL) which was thought to be due to malabsorption from prolonged *Cryptosporidium* infection. HLA-DQ2 and HLA-DQ8 were not tested, nevertheless, presence or absence of these autosomal dominant traits do not confirm or rule out celiac disease.

**Fig. 1.**
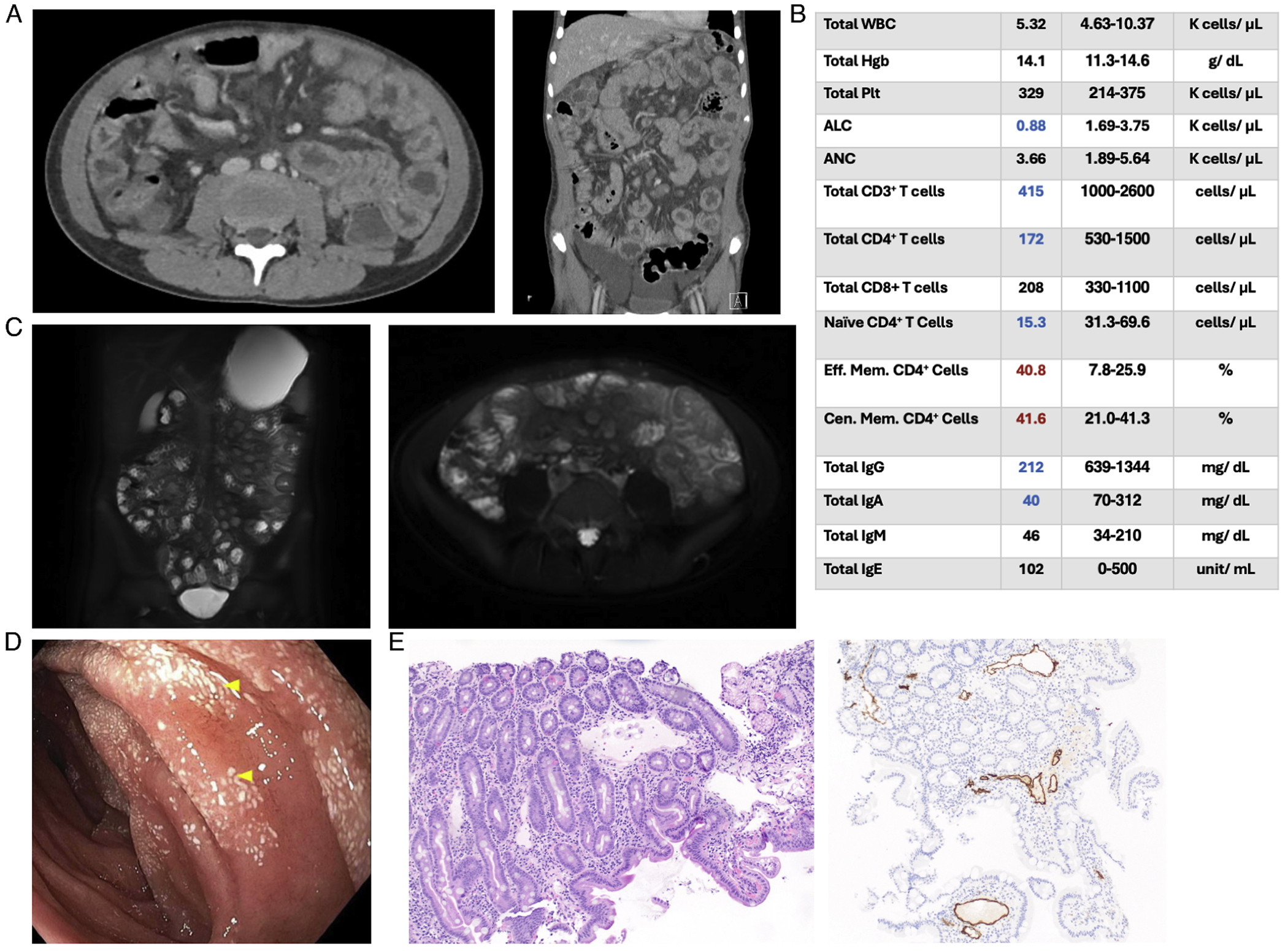
Initial diagnostic work-up. (A) Axial and coronal contrast-enhanced CT images demonstrate diffuse, concentric wall thickening of the small bowel and small amount ascites. (B) Initial immunology laboratory results obtained prior to the initiation of any immunomodulatory treatment. Values in blue are low while those in red are elevated. (C) Axial and coronal T2-weighted MR images demonstrate diffuse, concentric wall thickening of the small bowel. (D) GI endoscopy of the duodenum illustrating the gross appearance of PIL through the patches of prominent, white-tipped villi (yellow arrows). (E) Histopathology from endoscopy biopsy samples. Left: hematoxylin and eosin (H&E), 100x magnification: dilated lymphatics appear as “empty spaces” within the lamina propria. Right: stained with D2-D40 immunostain, 100x magnification: dilated lymphatics are highlighted in brown, confirming lymphatic identity.

Abdominal ultrasound showed marked bowel wall thickening (*not shown*) and CT of the abdomen and pelvis revealed diffuse enterocolitis and mesenteric lymphadenopathy (Fig. 1A). After consultation with the Pediatric Infectious Disease team, the patient was treated with a three-day course of nitazoxanide, leading to temporary symptom resolution. Follow-up labs showed improvement in albumin and protein levels, and normalization of both TTG IgA and fecal calprotectin. Given that the TTG IgA normalized despite continued gluten consumption by the patient, a diagnosis of celiac disease was felt to be unlikely. The recurrence of *Cryptosporidium*-associated diarrhea prompted a second, prolonged fourteen-day course of nitazoxanide. Repeat CT imaging three months later demonstrated resolution of ascites and improvement in bowel wall thickening, but persistent lymphadenopathy remained. Given the recurrent infection, the patient was referred to our outpatient Immunology clinic for assessment.

In the immunology clinic, immunophenotyping revealed combined immunodeficiency, characterized by severe hypogammaglobulinemia (total IgG: 212 mg/dL) and significant T cell lymphopenia (CD4^+^ count: 172 cells/μL; CD8^+^ count: 208 cells/μL) (Fig. 1B). The severe hypogammaglobulinemia required replacement, especially with a noted recurrent infection. The patient was started on subcutaneous immunoglobulin replacement therapy and trimethoprim/sulfamethoxazole prophylaxis.

Genetic evaluation, including a comprehensive primary immunodeficiency panel and whole exome sequencing, identified a heterozygous pathogenic deletion of exons 2–4 in the ADA2 gene, with the deletion containing the initiator codon. This gene is associated with deficiency of adenosine deaminase 2 (DADA2), an autoinflammatory disorder with varying phenotypes including vasculitis, immunodeficiency, and hematologic abnormalities. The DADA2 disorder generally follows an autosomal recessive inheritance pattern even though some missense variants are suspected to have a dominant negative effect [5]. The patient’s peripheral blood ADA2 level was 2 U/ L, expectedly at the low end of the carrier range. A heterozygous deletion as found in this patient would not be expected to function in a dominant-negative way and thus be disease-causing as there is no correlation between ADA2 activity and disease severity [5,6]. Lastly, the patient’s mother is a carrier of the same variant and presents as healthy. Considering all these aspects, we therefore consider this variant unlikely to be the cause of his presentation.

Despite the treatment with subcutaneous immunoglobulin replacement therapy and trimethoprim/sulfamethoxazole prophylaxis, immune parameters remained poor (total IgG: 515 mg/dL; CD4^+^ count: 150 cells/μL; CD8^+^ count: 153 cells/μL). Due continued occasional nausea and vomiting, his gastroenterologist evaluated for possible protein-losing enteropathy. Consistent with this a fecal alpha-1 antitrypsin measurement was above the level of detection of 1.13 mg/g (0.00–0.50 mg/g), on two separate occasions, with persistent hypoalbuminemia (2.2 g, range 3.1–4.8 g/dL) and hypoproteinemia (total protein 3.1 g (range 6.1–8.0 g/dL). Fecal calprotectin remained mildly elevated at 58.9 mg/kg. An MR enterography with contrast found again diffuse small bowel wall thickening as well as lymphadenopathy (Fig. 1C) and the patient was referred to undergo esophagogastroduodenoscopy (EGD).

EGD with duodenal biopsies, together with the laboratory values, radiographic imaging and clinical presentation noted above, confirmed the diagnosis of PIL (Fig. 1D, E). Other causes of protein-losing enteropathy were considered such as infectious (normal infectious studies including *Helicobacter pylori*), cardiac (normal echocardiogram, EKG and cardiology evaluation), IBD (colonoscopy with biopsies not consistent) and malignancy (cross-sectional imaging did not identify a mass). Fecal pancreatic elastase was not decreased indicating no exocrine pancreatic insufficiency (>800 μg/g, range: ≤ 100 μg/g). MR lymphangiography (only performed after diet initiated as below), which confirmed no abnormalities in the central conducting lymphatic channels in the chest, abdomen, and pelvis. A lymphoscintigraphy or MR lymphangiography of the lower extremities was not performed since the patient had no lower extremity swelling concerning lymphedema.

**Fig. 2.**
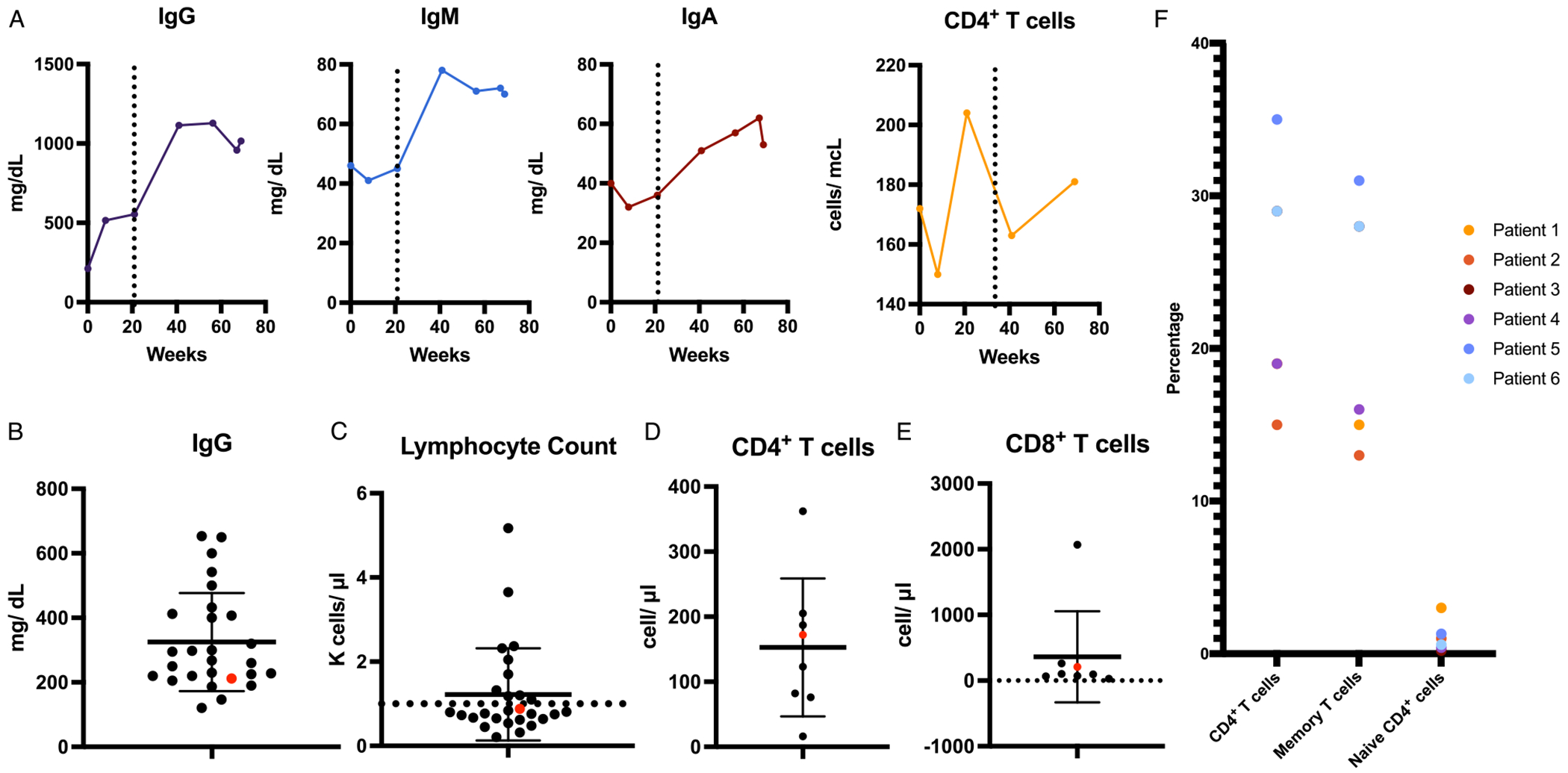
trajectory of immunophenotyping results. (A) Patient’s IgG, IgA, IgM, and CD4^+^ T cell levels over time. The dotted line represents when the dietary intervention was initiated. (B) Comparison of our patient’s IgG levels (red) to twenty-six cases in the literature [2,3,9–22]. (C) Comparison of our patient’s lymphocyte count (red) to twenty-six reported cases [2,3,9,10,12,14,17–19,21–26]. The dotted line indicates a value of 1000 K/ μL as the usual lower normal range. (D) Comparison of our patient’s CD4^+^ T cell count and (E) CD8^+^ T cells count (red) to seven other patients in literature [3,10,12,14,19]. (F) T cell subset percentages for our patient (Patient 1) and five other cases from literature [14,18,27].

The patient was promptly started on a new diet consisting of a strict low-fat diet (12 *g* per day), high-protein intake (75 −125 *g* per day), supplementation with fat-soluble vitamins (A, D, E, K), and daily medium-chain triglyceride oil. At follow-up after diet initiation, the patient reported significant clinical improvement. He experienced only 1–2 episodes of diarrhea per month, with resolution of bloating, abdominal pain, nausea, vomiting, and peripheral swelling. Immunologically, his total IgG level increased to 1114 mg/dL, although his T cell lymphopenia remained unchanged (Fig. 2A).

### Literature review

To compare our case to existing reports and assess the general knowledge of PIL as a cause of secondary immunodeficiency we reviewed the medical literature. We performed a search of the MEDLINE database (accessed via PubMed) and Google Scholar, with broad search terms to find any study referencing intestinal lymphangiectasia or protein-losing enteropathy with no limit to the time period of the study. Exclusion criteria included intestinal lymphangiectasia due to a stated specific secondary cause. Subsequently, studies found were screened for the availability of immunophenotyping laboratory values or reported opportunistic infections. Given the limited number of studies identified meeting even these broad criteria, all remaining were included to the best of our ability.

The underlying cause of PIL remains unknown, however, some evidence suggests the pathogenesis arises from altered expressions of regulatory molecules in the duodenal mucosa [7,8]. In a review of 84 PIL cases by Wen *et al*., the disorder was found to primarily affect children, with 53% of cases presenting under two-years old. Hypoproteinemia is a clear feature of the disorder found in 83% of children with PIL and 85% of adults. With respect to clinical manifestations, edema, diarrhea, and ascites were the most prevalent, affecting 83%, 58%, and 50% of children, and among adults, these manifestations were observed in 72%, 67%, and 31% of cases, respectively [2].

To assess the prevalence of immunodeficiency in this patient population, we searched the literature for studies of primary intestinal lymphangiectasia that included data on key immune parameters. We found that 66% of reported cases had a total IgG below 400 mg/dL, although some of these include infants with potentially physiologically low IgG at the time of measurement (Fig. 2B). Similarly, we found lymphopenia commonly reported, with 66% of reported cases noting an absolute lymphocyte count below the normal range (Fig. 2C). In the 8 patients with additional immunophenotyping reported, we found that when lymphocyte counts are depleted, this loss was predominantly in CD4^+^ T cells, with CD8^+^ T cells, B cells and NK cells less affected (Fig. 2D, E and *not shown*). 6 patients had additional subtyping of naïve and memory CD4^+^ T cells reported. As shown in Fig. 2F, specifically naïve CD4^+^ T cells are highly decreased in these PIL patients, with retention of memory and effector T cells, likely responsible for some baseline protection against infection [14,27]. One hypothesis regarding the reason for specific naïve T cell loss in these patients is that naïve cells recirculate between blood and secondary lymphoid organs, including via lymphatic vessels [14]. With the increased lymphatic pressure induced by PIL, leakage of these cells into the gastrointestinal lumen occurs, leading to the loss. Memory T cells, however, commonly travel by blood to nonlymphoid tissues and peripheral sites of inflammation in addition to lymph nodes[14]. and are thus comparatively protected against intestinal loss.

Despite the fact, as we detail above, that many patients present with immunodeficiency as measured via laboratory testing, PIL has not been largely associated with opportunistic infections [1]. Our patient specifically experienced a recurrent *Cryptosporidium* infection. Two other PIL patients also experienced this opportunistic infection, one at the age of twelve and the other at one year old [10]. Limited information is available in regard to the susceptibility of PIL patients to *Cryptosporidium*. Studies indicate that the *Cryptosporidium* antigen is acquired by dendritic cells in the gut and trafficked to lymph nodes to prime naïve T cells [28]. A functional study by Fuss *et al*. further found that circulating T cells in patients with IL skewed toward a T helper 2 (Th2) cell cytokine pattern marked by reduced IFN-y/ IL-2 production and increased IL-4 production [27]. This is notable as *Cryptosporidium* promotes a Th1-like response dominated by IFN-γ production[28]. Other infections reported by patients with IL included cryptococcal meningitis, pneumococci meningitis, pertussis, recurrent ENT infections, and several patients with cutaneous warts [10,19,20,29–31]. We speculate that PIL patients may be specifically prone to these infections given their decreased naïve CD4^+^ T cells, perhaps with an associated diminished Th1 response, although that remains to be determined.

In addition to susceptibility to infection, a connection between PIL and lymphoma, while poorly understood, has been reported. In one review of 50 PIL patients, 3 had lymphoma, whereas in a review by Wen et al. of 84 cases 5% developed lymphoma [2,23]. Another study examined 10 studies, and including their patient, provided 14 cases of PIL-associated lymphoma. The time from PIL onset to lymphoma detection was 19.14 ± 12.29 years on average, finding that the lymphoma may present as an urgent clinical presentation with abdominal pain [2,23]. One theory is that depression of cell-mediated immunity by CD4^+^ T cells prevents proper regulation of B cells leading to uncontrolled proliferation [29].

## Discussion

In our case study and review of the literature, we find that PIL patients can present with significant immune abnormalities that can influence infection risk and long-term complications. Patients with PIL may present with decreased IgG and naïve CD4^+^ T cells, increasing susceptibility to opportunistic infections. Infections found in these patients include *Cryptosporidium* as seen in our case, as well as other infections where immunoglobulin and/or a CD4^+^ T cell response is important such as encapsulated bacterial infections and cutaneous warts. Clinicians should furthermore be aware that a correlation between PIL and lymphoma has been suggested. A key limitation of our literature review and interpretations is the heterogeneity of the comparison cohort (infants through adults, different PIL severities, limited availability of laboratory values).

In terms of treatment options for this condition, the strict low-fat, high-protein diet intervention was shown to have the largest impact in our patient and should act as the first line of treatment for PIL. The efficacy of the diet is thought to arise from lessening the pressure within lymphatic vessels. Since long-chain triglycerides metabolism utilizes the lymphatic system, it is believed to increase pressure and therefore leakage of both chylomicron fat and plasma protein [2,4]. MCTs bypass the intestinal lacteals, getting absorbed by passive diffusion along the gastrointestinal tract directly into the portal venous system. Therefore, by limiting LCT intake and supplementing with MCT, the pressure within the lymph is lessened and leakage through the intestinal tract can be modulated [1,4]. The diet intervention that proved efficacious for our patient, as seen in Fig. 2A, proving consistent with the finding of intervention showing therapeutic improvement in 85% of child cases, however, only 39% of adults [2]. This difference may be explained by children being more adaptable both in lifestyle and intestinal physiology in comparison with adults [2].

Diet initiation if effective will show gradual improvement in immune markers as in our patient and others, but in cases with clear immune deficiency, initiation of subcutaneous IgG should be considered. Subcutaneous IgG, rather than intravenous IgG, was found to provide a steadier state of serum IgG than monthly IV bolus dosing [32]. Depending on the scale of T cell lymphopenia, patients should also be placed on trimethoprim/sulfamethoxazole prophylaxis.

Outside of these first-line treatments, which second-line therapy best suits a patient largely depends on the extent and region of the lymphangiectasia. Embolization or surgery should be considered if the abnormal lymphatics are localized [4]. There have been several successful cases and permanent remission can be reached. For more extensive and pervasive lymphangiectasia, octreotide or sirolimus could prove efficacious. Octreotide suppresses gastrointestinal motility causing decreased intestinal absorption of fats. However, since its mechanisms being closely tied to the gastrointestinal system it is hypothesized to be optimal for patients with only intestinal involvement of the abnormal lymphatics [4]. Patients with lymphangiectasia exclusive to the intestines have reported response to octreotide without recurrence after discontinuation. Sirolimus acts on lymphatic endothelial cells inducing changes in mTOR signaling by blocking expression of vascular endothelial growth factor (VEGF) which is a key regulator in lymphangiogenesis. However, sirolimus’ actions are not able to be restricted and can affect any lymphatic vessel in the body [4].

In conclusion, PIL is a potential treatable cause of hypogammaglobulinemia and secondary combined immunodeficiency. Identifying treatable forms of immunodeficiency is essential and immune parameters should be reviewed in all patients presenting with protein-losing enteropathy (PLE), especially when opportunistic infections are present. Conversely, in patients with unexplained hypogammaglobulinemia, especially if lymphopenia is also present, forms of PLE including PIL should be considered. Interdisciplinary management by gastroenterology, nutrition, and immunology is highly recommended for this complex and rare condition.

## Data Availability

Data will be made available on request.
